# Brain-Specific Cytoskeletal Damage Markers in Cerebrospinal Fluid: Is There a Common Pattern between Amyotrophic Lateral Sclerosis and Primary Progressive Multiple Sclerosis?

**DOI:** 10.3390/ijms160817565

**Published:** 2015-07-31

**Authors:** Ahmed Abdelhak, Andreas Junker, Johannes Brettschneider, Jan Kassubek, Albert C. Ludolph, Markus Otto, Hayrettin Tumani

**Affiliations:** 1 Department of Neurology, Ulm University, Oberer Eselsberg 45, 89081 Ulm, Germany; E-Mails: ahmed.abdelhak@uni-ulm.de (A.A.); jbre@mail.med.upenn.edu (J.B.); jan.kassubek@uni-ulm.de (J.K.); albert.ludolph@rku.de (A.C.L.); markus.otto@uni-ulm.de (M.O.); 2 Institute of Neuropathology, University Hospital Göttingen, Robert-Koch-Str 40, 37075 Göttingen, Germany; E-Mail: andreas.junker@med.uni-goettingen.de

**Keywords:** primary progressive multiple sclerosis (PPMS), amyotrophic lateral sclerosis (ALS), neurofilaments, biomarker, tau, tubulin

## Abstract

Many neurodegenerative disorders share a common pathophysiological pathway involving axonal degeneration despite different etiological triggers. Analysis of cytoskeletal markers such as neurofilaments, protein tau and tubulin in cerebrospinal fluid (CSF) may be a useful approach to detect the process of axonal damage and its severity during disease course. In this article, we review the published literature regarding brain-specific CSF markers for cytoskeletal damage in primary progressive multiple sclerosis and amyotrophic lateral sclerosis in order to evaluate their utility as a biomarker for disease progression in conjunction with imaging and histological markers which might also be useful in other neurodegenerative diseases associated with affection of the upper motor neurons. A long-term benefit of such an approach could be facilitating early diagnostic and prognostic tools and assessment of treatment efficacy of disease modifying drugs.

## 1. Introduction

Multiple Sclerosis (MS) is the most common autoimmune disease of the central nervous system (CNS) in young adults affecting about 30 in 100,000 (global prevalence according to WHO 2008, available online: http://www.who.int/mental_health/neurology/Atlas_MS_WEB.pdf). The majority of MS-patients face the relapsing remitting form of the disease, in which the attacks are usually a sign of acute exacerbation of the inflammation [1]. After an average period of 19.1–21.4 years, about one third of patients progress to the secondary phase of the disease, which is characterized by slowly accumulating disability with or without acute exacerbations [2]. However, about 11%–18% of the patients have primary progressive multiple sclerosis (PPMS) with continuous slowly accumulating disability [3], mainly caused by the irreversible loss of axons [1]. This neurodegenerative process is believed to be closely associated with inflammatory activity especially in the progressive forms of the disease [4]. Yet, the current anti-inflammatory therapies used in relapsing remitting multiple sclerosis (RRMS) fail to alter the progression of both the primary and secondary forms of the disease [5].

Another chronic neurological disease associated with both neurodegeneration and neuroinflammation is amyotrophic lateral sclerosis (ALS), which is the most common motor neuron disease in adults with an incidence of 1–2 cases per 100,000, leading to death after a disease duration of 3–5 years [6,7,8]. ALS is characterized by degeneration of the primary and secondary motor neurons with subsequent respiratory failure due to muscle wasting. Its pathophysiology is complex and multifactorial involving several genetic factors which cause protein aggregation and formation of ubiquitin-positive, tau- and α-synuclein-negative TDP-43 (TAR DNA-binding protein 43), FUS (fused in sarcoma) inclusions [9]. Oxidative stress and glutamate excitotoxicity are also presumed to play an important role in the degeneration of the motor neurons [10]. PPMS and ALS, although two different etiologic entities, share some common pathophysiological pathways involving axonal death, apoptosis, and gliosis occurring already in preclinical stages. Degeneration of the long tracts in the spinal cord with clinical signs of upper motor neurons (UMN) occur in PPMS patients [11] as well as in ALS [7] associated with progressive spinal cord atrophy in both diseases [12,13]. Moreover, other diseases involving the long tracts of the spinal cord like hereditary spastic paraplegia (HSP) and primary laterals sclerosis (PLS) share similar clinical features with PPMS and classical ALS [14,15] and may challenge differential diagnosis [16,17]. Further studies regarding the mechanisms involved in these pathways could help to develop a common strategy to understand, prevent or at least slow the ongoing axonal degeneration and the resulting disability. Validation of CSF biomarkers and analyzing them in conjunction with other imaging markers of neurodegeneration (volumetry, diffusion tensor imaging and MR-spectroscopy) is a promising approach which could be helpful for establishing early diagnostic and prognostic tools, monitoring the progression and assessment of treatment efficacy of disease modifying drugs.

## 2. Pathophysiology of ALS

Since the first description of ALS about more than 100 years ago, the main pathophysiological mechanisms still remain unclear. In familial ALS (fALS) patients (about 10% of cases) many genes have been linked with the disease. c9ORF72 is currently considered the most frequent mutation in ALS causing nearly 40% of fALS and 7% of sporadic ALS (sALS) [18]. Other mutations include TAR DNA binding protein (TARDBP), fused in sarcoma (FUS), superoxide dismutase-1 (SOD1) and, less commonly, mutations affecting other proteins like optineurin (OPTN), ubiquilin-2 (UBQLN2) and ataxin-2 (ATXN2) and recently TANK-binding kinase 1 (TBK1) play a role in some familial cases of ALS [19,20]. All sALS and fALS cases carrying mutations in TARDBP have characteristic transactive response DNA binding protein 43 kDa (TDP-43) aggregation [21]. Few other cases show FUS or SOD1 aggregation in patients carrying FUS or SOD1 mutations, respectively [22,23]. TDP-43 has a wide range of physiological functions including an important role in RNA splicing, miRNA synthesis, RNA transport and many other RNA metabolic processes [24]. Most TDP-43 mutations affect the C-terminal glycine-rich domain of the protein, indicating that altering the function of this domain alone may be sufficient to cause neurodegeneration [25]. Post-translational modifications of TDP-43 include phosphorylation, ubiquitylation and C-terminal truncation [21] Whether TDP-43 aggregation is the direct cause of cell death or just the result of other neurotoxic processes and which effect it has on the viability of neurons remain unclear [21]. However, given the wide range of physiological functions, loss of nuclear TDP-43 may be sufficient to cause neurodegeneration [26]. Like TDP-43, FUS is a RNA binding protein which plays a role in splicing, miRNA processing and transport of mRNA between the nucleus and the cytoplasm [27,28]. Most mutations of FUS affect the nuclear localization sequence (NLS), which is essential for the shuttling of the protein between the nucleus and the cytoplasm [22,24]. The mutated FUS is, in contrast to TDP-43, neither phosphorylated nor ubiquitinated [19]. Further pathophysiological aspects of these different proteinopathies include prion-like pathologies, protein sequestration, stress granule formation and dysfunction of protein degradation pathways [19]. Degeneration of the motor neurons is followed by an inflammatory reaction with gliosis and accumulation of activated microglia and astrocytes with the production of cytotoxic molecules and inflammatory cytokines like TNF-α, and IL-1β [29]. Indeed, glial cells play an important role in the pathophysiology of the ALS. Because of a deficient astrocyte-specific glutamate transporter (GLT-1) or excitatory amino acid transporter-2, (EAAT2), the astrocytes fail to clear up the glutamate leading to exacerbation of the glutamate excitotoxicity [30]. Moreover, the role of astrocytes and microglia is supported by the observed increase in production of reactive oxygen species, Nitric oxide (NO) and interferon-γ [30,31]. The role of microglia is more evident in the late stages of the disease [32]. Recently, the oligodendrocytes have been found to be involved through loss of monocarboxylate transporter 1 (MCT1) resulting in altered metabolism and axonal degeneration [33]. Furthermore, emerging evidence shows an increase of dendritic cells, CD-4+ and CD-8+ T-Lymphocytes, in proximity to dying motor neurons [34]. Both mutant TDP-43 [35] and extracellular mutant SOD1 [36] can induce a local inflammatory reaction. The anti-inflammatory drug Lenalidomide increased survival and improved motor performance in SOD1 mutated mice in correlation with decreased expression of TNF-α, and IL-1β [35] highlighting the role of inflammation in the pathophysiology of the disease.

Over the last years, a better understanding of the dynamics of ALS has evolved by work on morphological characterization of the neurodegenerative spreading through the nervous system. Postmortem examination of the distribution of TDP-43 aggregation in the brain and spinal cord revealed four distinct stages of the pathology (Braak staging). TDP-43 aggregates appear in the agranular motor cortex, brainstem motor neurons and α-motor neurons in the spinal cord (stage 1). The disease progresses afterwards affecting prefrontal neocortex and reticular formation (stage 2) followed by postcentral gyrus and striatum (stage 3) and ends by involvement of the anteromedial temporal lobe and hippocampus (stage 4) [37]. The TDP-43 pathology starts in the central nervous system as a focal (e.g., in thoracic or lumbar spinal cord segments), bifocal or multifocal process and propagates to other areas by transneuronal signaling or axonal transport either in a direct neuroanatomic pattern or through connected neuronal networks [38,39].

## 3. Pathophysiology of Progressive Multiple Sclerosis (MS)

The pathological hallmark of MS is inflammation induced demyelination and subsequent axonal loss [40,41], which may be initially accompanied by remyelination in part of the lesions [42,43]. Pathological studies revealed different types of plaques depending on the stage of the inflammatory reaction (classic active plaques, slowly expanding lesions, inactive plaques and remylinated shadow plaques) with a predominance of the first in RRMS and the second in progressive types of MS [44]. Histopathological examination of acute MS lesions revealed different patterns of tissue injury for which different causal mechanisms are presumably responsible [45,46]: T cell infiltrates and macrophage-associated tissue injury (pattern 1); antibody and complement-mediated immune reactions against cells of the oligodendrocyte lineage and myelin (pattern 2); hypoxia-like injury, resulting either from inflammation-induced vascular damage or macrophage toxins that impair mitochondrial function (pattern 3); and a genetic defect or polymorphism resulting in primary susceptibility of the oligodendrocytes to immune injury (pattern 4) [45]. The inflammatory cell infiltrates include T-cells with a predominance of CD-8+ T-lymphocytes, B-lymphocytes and activated macrophages [47].

A characteristic feature of late stages of the disease is the presence of intact blood-brain barrier (BBB). This is also apparent through the absence of gadolinium enhancing lesions. Nevertheless, there is a diffuse ongoing inflammatory reaction in the CNS tissue in particular in chronic MS, which is not necessarily restricted to lesions but also occurs in the white or grey matter apart from lesions [48]. Thus, a considerable axonal damage is also apparent in non-demyelinated areas [49]. Diverse mechanisms could be involved in the process of axonal injury such as cytotoxic T cells, macrophageal enzymes, microglial and astrocytic activation, oxidative stress, altered axonal ion homeostasis, mitochondrial dysfunction and iron accumulation [50,51].

## 4. Physiology of Cerebrospinal Fluid (CSF) and Its Relationship with Brain Specific Proteins in CSF

CSF originates mainly from the choroid plexus and the interstitial fluids of the brain and meninges. About 80% of the CSF proteins derive from the blood and enter the CSF mainly by passive diffusion over the blood CSF barrier (BCB) [52]. Measuring the CSF-serum albumin ratio is a well-accepted marker of the CSF flow rate which is influenced by several factors such as barrier permeability, CSF production and elimination [53]. The blood-derived molecules continuously enter the CSF along its way through the subarachnoid space leading to a continuous increase in their concentration along the CSF-circulation moving away from the ventricles towards the caudal end of the CSF space. The concentration of the molecules originating from the brain tissue usually tends to decrease by moving away from the ventricles and simultaneous dilution by CSF components originating from blood [54]. Another important aspect regarding the concentration of some molecules is the topographic distribution of different brain pathologies. The frontal, parietal and occipital lobe are considered CSF-distant leading to limited ability of the CSF analysis as a mirror for the pathological changes in contrast to diseases of meninges, temporobasal region and the spinal cord [55]. Concentration of brain specific proteins in lumbar CSF can be elevated in various pathologies of the brain independent of etiology (Alzheimer disease, ALS, meningoencephalitis, MS). In addition, several additional factors may influence concentration of CSF proteins and have to be considered when studying CSF parameters such as (a) circadian variation; (b) volume of sampled CSF and (c) rostro-caudal concentration gradient [56,57].

## 5. CSF Markers of Neuroaxonal Damage

### 5.1. Neurofilaments

Neurofilaments are one of the class IV intermediate filaments and a major structural element of the axons and dendrites of the neurons. They are formed from four subunits. neurofilament light-chain (NfL) of 68 kDa, which form a backbone for the neurofilament medium-chain (NfM) of 150 kDa and neurofilament heavy-chain (NfH) of 190–210 kDa to assemble [57]. Each subunit is formed from two double stranded alpha helical structure [58]. The fourth subunit is either α-internexin in the CNS or peripherin in the peripheral nervous system (PNS) [59]. Complete neurofilaments (10 nm) interact with actin microfilaments and microtubules to form the cytoskeleton of large myelinated axons, which plays an essential role in stabilizing the axon structure and maintain axonal transport of nutrients and organelles [60]. Neurofilaments undergo extensive phosphorylation, especially at the C-terminus of NfH and NfM by cofactor-dependent and cofactor-independent kinases, which is essential for its crosslinking and stabilization of the axonal cytoskeleton [61]. The degree of phosphorylation determines the diameter of the axon [62]. Highly phosphorylated NfH is only found in axonal neurofilaments and is partially resistant to proteases in contrast to NfL which is also present in the cell bodies and dendrites [63]. Consequently, highly phosphorylated NfH may be considered as a surrogate marker for axonal damage [64]. Neurofilaments are generally metabolically stable during their transport along the axons whereas dephosphorylation and degradation mostly take place at the synapses [65].

#### 5.1.1. Neurofilaments (NF) in ALS

The role of neurofilaments in ALS have been intensively studied in the last 20 years. Munoz and colleagues have detected an abnormal accumulation of phosphorylated neurofilaments in cell bodies of anterior horn motor neurons of the spinal cord [66]. Mutations leading to polymorphism in the 43 Lys-Ser-Pro (KSP) motifs of the neurofilament gene were found in some sporadic ALS patients and could play a role in the pathophysiology of ALS [67]. Measuring the CSF level in different studies showed similar results. CSF level of NfL and NfH was significantly higher when compared with healthy controls [68,69], ALS-mimics [70] and other neurological diseases [71,72] in all studies (Table 1). Higher levels of neurofilaments tend to be associated with rapid progression of the disease [69,71,72,73]. The levels are similar between sALS and fALS. However, patients with SOD1 mutation tend to show a relatively lower concentration, which could be explained by changes in NfL mRNA by mutant SOD1 [71]. Patients with predominant upper motor neuron lesion (UMNL)-signs have higher levels than patients with predominant lower motor neuron lesion (LMNL)-signs in all mentioned studies but one [73]. This study also revealed different levels according to site of onset of the disease, with a two to three fold higher level in patients with bulbar onset compared to those with spinal onset. However, this result was not statistically significantly different from results described in other studies, which did not show any difference. The levels were related to prolonged central conduction time in motor evoked potentials (MEP) [70]. No relation could be found with either the muscle power in medical research council sum score (MRCS) [69] or with changes in electromyography (EMG) [70]. The levels were similar in patients with and without riluzole therapy [69].

**Table 1:** Neurofilament cerebrospinal fluid (CSF) levels in amyotrophic lateral sclerosis (ALS) as compared to healthy controls, ALS-mimics and other neurological diseases.

Marker	Method	Sample Size	Finding	Correlation with Progression Rate	Reference
NfL	ELISA	12 ALS, 11 AD, 34 control	ALS > AD	not reported	[68]
	ELISA	60 sALS, 19 fALS, 206 OND	ALS > reference > control	yes	[71]
	ELISA	32 ALS, 26 ALS-mimic	ALS > ALS-mimic	not reported	[70]
	ELISA	37 ALS, 25 CIDP, 21 OND	ALS > CIDP and OND	yes	[72]
	ECL	38 ALS, 20 control	ALS > control	not reported	[74]
NfH	ELISA	ALS 69, 33 Control	ALS > control	yes	[69]
	ELISA	32 ALS, 26 ALS-mimic	ALS > ALS-mimic	not reported	[70]
	ECL	50 ALS, 73 controls	ALS > control	not reported	[75]
	ELISA	20 ALS	Higher level is associated with rapid progression	yes	[73]

NfL: Neurofilament Light-chain; NfH: Neurofilament Heavy-chain; ELISA: Enzyme-linked immunosorbent Assay; AD: Alzheimer’s disease; ALS: Amyotrophic Lateral Sclerosis; fALS: Familial ALS; OND: Other Neurological Diseases; CIDP: Chronic Inflammatory Demyelinating Polyneuropathy; ECL: Electrochemiluminescence.

#### 5.1.2. Neurofilaments (NF) in Primary Progressive Multiple Sclerosis (PPMS)

NfL and NfH levels are increased in CSF of MS-patients when compared with healthy controls or patients with other neurological diseases (Table 2). However, contradicting results about the levels in subtypes of MS have been reported, since CSF-neurofilament levels in progressive multiple sclerosis (PMS) were either higher [57,76] or lower [77] or equal [59,78] to levels in RRMS. The same could be found regarding the relation to clinical severity (Expanded disability status scale, EDSS). NfL shows a statistically significant correlation with inflammatory markers like the chemotactic cytokine CXCL13 and myelin basic protein (MBP) [77] but not to cell count or albumin-quotient [59]. Higher levels of NfH^SMI35^ at baseline tend to be associated with EDSS progression at follow up [76]. Similarly, high NfL at baseline was significantly correlated with multiple sclerosis severity scale (MSSS) at follow up [79]. Moreover, patients with progressive MS (PMS) from onset tend to have a higher NfL at baseline than RRMS patients [57].

**Table 2:** Neurofilament CSF levels in primary progressive multiple sclerosis (PPMS) as compared to healthy controls, relapsing remitting (RRMS) and secondary progressive multiple sclerosis (SPMS) and other inflammatory- and non-inflammatory neurological diseases.

Marker	Method	Sample Size	Finding	Correlation with Disability	Reference
NfL	Dot Blot	16 PPMS, 13 SPMS, 6 PPMS, 36 IND and NIND	PMS > RRMS > control	yes	[57]
	ELISA	21 RRMS, 20 SPMS, 10 PPMS	Non-measurable	no	[78]
	ELISA	65 RRMS, 10 SPMS, 20 PPMS	Sig. correlation between NfL and MSSS at follow up	not reported	[79]
	ELISA	33 RRMS, 7 SPMS, 1 PPMS	PMS > RRMS > OND	yes	[80]
	ELISA	40 SPMS, 21 PPMS, 26 RRMS, 20 NIND	RRMS > PMS > NIND	not reported	[77]
	ELISA	38 RRMS, 25 SPMS, 23 PPMS, 62 CIS, 72 healthy control	RRMS/SPMS/PPMS > control	no	[59]
NfH	In-House ELISA technique	23 SP/PP MS, 11 RRMS, 318 NIND	NfH^SMI35^ PMS > RRMS	yes	[76]
	ELISA	21 RRMS, 20 SPMS, 10 PPMS	RRMS = SPMS = PPMS > controls	no	[78]

PPMS: Primary Progressive Multiple Sclerosis; SPMS: Secondary Progressive Multiple Sclerosis; IND: Inflammatory Neurological Diseases; NIND: Non Inflammatory Neurological Diseases; PMS: Progressive Multiple Sclerosis; RRMS: Relapsing Remitting Multiple Sclerosis; MSSS: Multiple Sclerosis Severity Scale; CIS: Clinically Isolated Syndrome.

### 5.2. Tau-Protein (Tau)

Tau proteins belong to the family of microtubule associated proteins. They are highly expressed in neurons and mainly found in axons. There are six tau isoforms due to alternative mRNA splicing [78]. The molecular weight varies between 50 and 75 kDa. Tau proteins play a very important role in microtubule assembly, bundling and stabilization. Typically, phosphorylation of Tau is an important factor regulating its function, however, phosphorylation of some residues could decrease or completely abolish its ability to bind to microtubules. Hyperphosphorylation of Tau leads to its accumulation and the formation of neurofibrillary tangles (NFT) which characterize many neurodegenerative diseases such as Alzheimer disease, Pick’s Disease, progressive supranuclear palsy (PSP) and corticobasal degeneration (CBD) [81,82].

#### 5.2.1. Tau-Protein (Tau) in Amyotrophic Lateral Sclerosis (ALS)

Measurement of CSF-Tau in ALS patients showed inconsistent results. There were no differences between ALS patients, healthy controls [83,84] and 4R-tauopathy like progressive supranuclear palsy (PSP) [85] (Table 3). However, some studies showed higher level compared to healthy controls [69,86]. CSF Tau was not related to the site of onset (bulbar *vs*. spinal) in all studies. One study found a relationship between UMN-Score and CSF Tau. The same study reported that patients in earlier disease stages exhibit a higher level than those with advanced disease [86].

**Table 3:** CSF levels of total Tau-protein (t-Tau) in Amyotrophic Lateral Sclerosis (ALS) as compared to healthy controls and other diseases associated with 4-Repeat-Tauopathy (4R-Tau).

Marker	Sample	Finding	Relation to Clinical Severity	Reference
t-Tau	20 sALS, 20 controls	70% of ALS have high CSF-tau compared to controls	no	[86]
	18 sALS, 75 control	ALS = control	no	[83]
	67 sALS, 2 fALS, 33 control	ALS > control	no	[69]
	57 sALS, 110 controls	ALS = controls	no	[84]
	51 ALS, 23 4R-tau, 23 control	ALS = 4R-Tau = control	yes	[85]

t-Tau: total Tau; sALS: Sporadic ALS; 4R-tau: 4-Repeat-Tauopathy.

#### 5.2.2. Tau-Protein (Tau) in Primary Progressive Multiple Sclerosis (PPMS)

Measurement of total Tau in CSF of PPMS patients provides, similar to RRMS, contradicting results among the different studies (Table 4). CSF-Tau was elevated in PPMS patients in comparison to controls without a significant difference when compared to other RRMS/SPMS (secondary progressive multiple sclerosis) patients [87,88,89]. Other studies showed that CSF-Tau did not significantly differ between PMS and other inflammatory neurological diseases, but was still higher than healthy controls [90]. Kapaki and colleagues found that highly elevated CSF-Tau was more common in PMS patients when compared to controls [87]. No difference between MS patients including PPMS and controls could be found in two studies [91,92]. A correlation between t-Tau and EDSS could not be established in any of the studies. Bartosik-Psujek found that concentrations of CSF-Tau were correlated with the IgG index in patients with inflammatory neurological diseases [90].

**Table 4:** CSF levels of total Tau-protein (t-Tau) in primary progressive multiple sclerosis (PPMS) as compared to healthy controls, relapsing remitting (RRMS) and secondary progressive multiple sclerosis (SPMS) and other inflammatory- and non-inflammatory neurological diseases.

Marker	Number	Finding	Correlation to Disability	Reference
t-Tau	15 RRMS, 11 SPMS, 10 PPMS, 17 ALS, 29 healthy controls	MS > control and ALS 72% of PMS having highly elevated tau compared to 27% of RRMS	not reported	[87]
	84 RRMS, 21 SPMS, 9 PPMS, 60 NIND, 79 IND	RRMS > IND > PMS > NIND	not reported	[90]
	43 defined MS, 20 CIS, 56 controls (OND)	MS = control	No	[92]
	50 CIS, 35 RRMS, 8 SPMS, 9 PPMS, 46 control	MS > control	No	[88]
	32 RRMS, 2 SPMS, 4 PPMS, 19 healthy controls	MS = Control	No	[91]
	30 RRMS, 9 SPMS, 6 PPMS, 38 healthy controls	MS > control	No	[89]

ALS: Amyotrophic Lateral Sclerosis; MS: Multiple Sclerosis; PPMS: Primary Progressive Multiple Sclerosis; SPMS: Secondary Progressive Multiple Sclerosis; OND: Other Neurological Diseases; RRMS: Relapsing Remitting Multiple Sclerosis.

### 5.3. Phosphorylated Tau-Protein (p-Tau)

#### 5.3.1. p-Tau in ALS

Levels of p-Tau were found to be significantly lower when compared either to healthy seniors or to patients with PSP [85]. In the same study, the reduction in the p-Tau to t-Tau ratio could distinguish ALS from 4R-tauopathy and from healthy controls with a good sensitivity and specificity (92% and 91.7%, respectively). Moreover, the low p-tau and reduced p-tau to t-tau ratio were related to clinical severity as defined by the revised ALS functional rating scale (ALSFRS-R), cognitive dysfunction in mini mental state examination (MMSE) and reduction of fractional anisotropy (FA) for the corticospinal tract and corpus callosum [85]. No correlation could be found with disease progression rate.

#### 5.3.2. p-Tau in PPMS

A recent histopathological study found abnormal accumulation of p-Tau in multiple cell types with glial predominance in PPMS [93]. There are many studies measuring the p-Tau in CSF from RRMS patients, however, none of them included any PPMS patients.

### 5.4. Tubulin and Actin

Another major components of the cytoskeleton are the microtubules and actin microfilaments (23 and 6 nm diameter, respectively). The microtubules usually consist of nine to 16 protofilaments, which are made up of alternating α and β tubulin monomers (each contain 450 a.a) [94]. There are six isoforms with predominant expression of classes II and III isotypes in the brain [95]. The actin microfilaments are formed mainly from two stranded helical polymers of actin (predominantly β or γ isoforms). They play an important role in supporting the neuronal cytoskeleton and transport of neurotransmitters along the neurites and their release at synapses [96,97]. In one study, the CSF-actin and tubulin levels were significantly higher in PPMS (*n* = 6) and SPMS (*n* = 13) when compared to RRMS (*n* = 16), healthy controls (*n* = 12) or non-inflammatory neurological diseases (*n* = 20) [57]. Another study showed significantly higher β-Tub II in PMS (*n* = 9) in comparison to OND (*n* = 18). Moreover, β-Tub III was higher in PMS than in RRMS [80]. In both studies, the levels strongly correlate with the EDSS score [57,80]. There are no sufficient data about the levels in ALS.

## 6. Discussion

**Figure 1:**
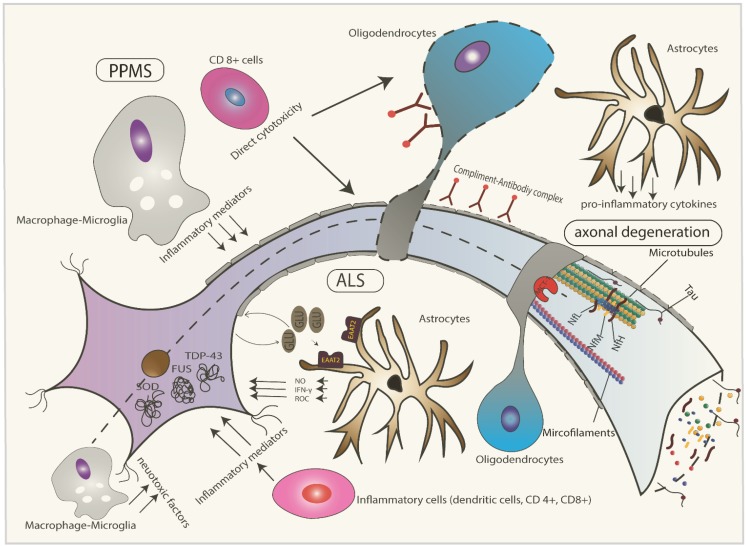
Main pathophysiological processes involved in Amyotrophic lateral sclerosis (ALS) and primary progressive multiple sclerosis (PPMS). ALS is characterized by accumulation of intracellular cytoplasmic aggregations (TDP-43, fused in sarcoma (FUS) and superoxide dismutase (SOD)) in the motor neurons. Glial cells play an important role in the pathophysiology through different pathways: defective astrocytic glutamate (GLU) uptake through excitatory amino acid transport (EAAT)-2 receptors resulting in glutamate excitotoxicity, defective lactate uptake by the oligodendrocytes through monocarboxylate transporter (MCT)-1 receptors and neurotoxic factors release from the microglial cells. Inflammatory cells like dendritic cells play a role through release of inflammatory mediators like interferon-γ (IFN-γ), reactive oxygen species (ROS) and nitric oxide (NO). In PPMS, however, the axonal loss is mediated by different mechanisms like macrophage and T-cell induced tissue damage, antibodies mediated damage of the myelin sheath and oligodendrocytes, hypoxic tissue injury and primary oligodendrocyte’s susceptibility. Both diseases result in axonal damage with increased levels of different markers in the cerebrospinal fluid (CSF) (total Tau-protein (t-Tau), phosphorylated Tau-protein (p-Tau), neurofilaments, actin and tubulin) and characteristic changes in magnetic resonance imaging (MRI) like brain atrophy and reduction of fractional anisotropy (FA).

Although ALS and PPMS are etiologically and pathophysiologically two different entities, they share some common end pathological landmarks, especially the axonal degeneration and release of different markers in the CSF (Figure 1) and (Table 5).

**Table 5:** Patterns of common CSF-Biomarkers of Axonal Damage: A comparison between Amyotrophic Lateral Sclerosis (ALS) and Primary Progressive Multiple Sclerosis (PPMS).

CSF-Markers of Axonal Damage	PPMS	Reference	ALS	Reference
Neurofilaments	PPMS > healthy controls	[75,76,77,78,79]	ALS > healthy controls	[68,69,70,71,72,73,74]
t-Tau	PPMS > or = healthy controls	[87,88,89,90,91]	ALS > or = healthy controls	[83,84,85,86]
p-Tau	PPMS < healthy controls	[93]	ALS < 4-repeat tauopathy	[85]

t-Tau: total Tau-protein; p-Tau: phosphorylated Tau-protein.

Neurofilament levels were elevated in almost all studies in both diseases. The high levels seem to be correlated with rapid clinical progression in ALS and PPMS. A relationship was found between NfH and both clinical and electrophysiological signs of UMN degeneration, justifying the use of NfH as a marker for degeneration of long axons in different neurological diseases. Interestingly, the correlation between NfL with some inflammatory markers like CXCL and CSF-Tau with IgG-index may support the hypothesis that neurodegeneration in multiple sclerosis is a direct result of continuous low-grade inflammatory activity and not a separate pathological process. Further evidence derives from recent histopathological studies showing a significant correlation between the inflammation and axonal degeneration in MS-Plaques with dying-out of the inflammation and degeneration phenomena in older-patients without other neurodegenerative diseases [4]. In contrast to neurofilaments, t-Tau measurement showed discrepancy between the levels in different studies. However, it does not universally correlate with disability. Although t-Tau is usually considered as an axonal marker, occurrence of highly elevated levels in diseases which primary involve the gray matter like Alzheimer’s disease (AD) and Creutzfeldt Jakob disease (CJD) could question its usefulness as a marker in diseases involving long tracts in the CNS. The role of P-tau, tubulin and actin as a biomarker in both ALS and PPMS require further study to determine their usefulness.

**Figure 2:**
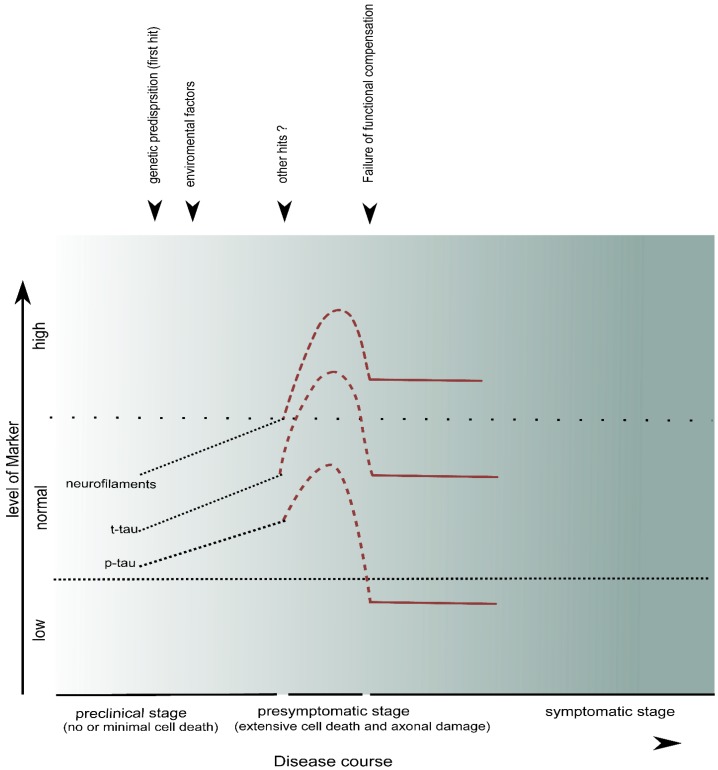
Dynamics of CSF-biomarkers in ALS. The long preclinical stage characterized by cellular changes like cytoplasmic vacuoles, mitochondrial changes and synaptic loss because of genetic predisposition (first hit) in addition to environmental factors and oxidative stress (other hits) would be associated with no or minimal cellular death and, thus, no or low level of marker in the CSF. In the presymptomatic stage, where extensive cell death and axonal damage takes place, there would be a massive release of CSF markers for neurodegeneration. The symptoms will appear when the damage exceeds the functional capacity of the CNS. In the symptomatic stage, the CSF levels of neurodegeneration markers do not show significant changes which can be explained by reduced volume of neuroaxonal structures. Less sensitive markers for axonal death like t-Tau will be even normal.

Attempts to correlate CSF NF-levels with clinical parameters in PPMS could be useful when related to individual functional system scores such as the expanded disability status scale (EDSS) or the multiple sclerosis functional composite (MSFC). One might expect to find a significantly higher NF-levels in patients with predominant pyramidal affection. However, a drawback in many of the mentioned studies in PPMS is trying to find a correlation between NF-levels and total EDSS without considering its single components such as motor functions. Other drawbacks were different techniques to measure NF-levels and small sample size in many studies. Measuring the biomarker levels in the CSF and their dynamics along the disease course of ALS may give an idea about the progress of the underlying pathological process even in the preclinical phase of the disease. This may have an impact on the early diagnosis and treatment of the disease. Examination of SOD1 mutant mice showed different changes in the cellular level like cytoplasmic vacuoles, vacuolated mitochondria and loss of synapses early in postnatal period long before developing clinical symptoms [98,99]. Similarly, in FUS and TDP-43 pathologies, the cellular changes like protein accumulation in the cytoplasm occur long before the cellular death takes place and a second hit or even multiple hits (environmental factors, oxidative stress) are needed to convert the rather soluble isolated punctate aggregation of the protein or within stress granule in the cytoplasm to the insoluble large cytoplasmic protein aggregations [22,28]. Furthermore, in one study, electrophysiological estimation of motor unit number (MUNE) in SOD1 mutation carriers revealed normal results in the asymptomatic stage when compared to age and sex matched family controls without the mutation. Another study showed normal EMG in two asymptomatic sALS patients about 18 month before the establishment of the diagnosis [100]. In summary, the study results suggest that a long preclinical period occurs where cellular changes accumulate with no or only minimal cell death followed by a relatively short presymptomatic stage, and where a rapid and widespread death of the motor neurons and axonal damage takes place. Only when the extent of neurological damage exceeds the functional plasticity of the nervous system will the symptoms appear [101]. We assume that these changes during the preclinical/presymptomatic period could be associated with changes in the CSF-biomarkers in ALS patients. Figure 2 demonstrates the dynamics of those markers over the course of the disease.

Furthermore, ALS patients form a heterogeneous group regarding the rate of disease progression. Younger patients and those with limb-onset disease show a slower disease progression [102]. Moreover, the disease progression was related in cross-sectional imaging studies with neuroimaging measures such as cortical thinning of precentral gyrus [103], temporal areas [104], bilateral and temporal areas [105] and lower fractional anisotropy (FA) and higher radial diffusivity in corticospinal tracts (CST) using diffusion tensor imaging (DTI) [106]. However, in longitudinal studies this FA reduction of the CST in the brain [106] and spinal cord [13], along with clinical upper motor neurons clinical scores, appears to be relatively constant over the disease course [106,107] unlike the rather progressive and widespread changes in cortical grey matter [106] and reduction of cord cross-sectional area [13].

**Figure 3:**
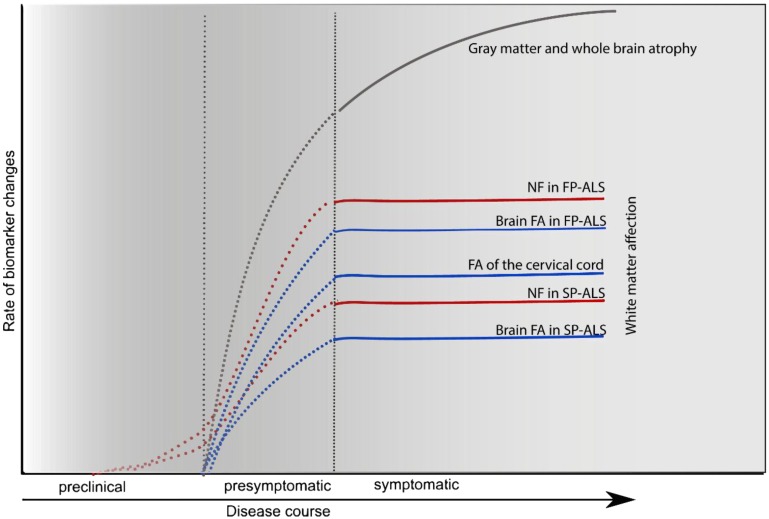
Dynamics of changes in the CSF and MRI-parameters of neurodegeneration over the course of the ALS. In different longitudinal imaging studies, the Fractional Anisotropy (FA) was relatively stable, but different between patients with fast (FP-ALS) and slow (SP-ALS) progressive ALS. The same studies showed progressive atrophy of the cortical grey matter and spinal cord in the symptomatic stages. This suggests that the major damage of corticospinal tract occurs in the presymptomatic stage of the disease. Accordingly, the rate of changes in FA and neurofilaments (NF) in the CSF would be greater in the presymptomatic stage rather than after appearance of the symptoms.

Accordingly, corresponding changes in the dynamics of neurofilaments and other markers of CST damage should be considered when comparing patients with fast (FP-ALS) and slow (SP-ALS) progressive ALS (Figure 3). Moreover, the above-mentioned concept casts doubt on the usefulness of therapies targeting the degeneration of the long tracks in symptomatic patients and should direct the research towards developing therapies targeting this progressive grey matter degenerative process and refining its measurement methods to be used as an endpoint in different clinical studies.

The comparison of different UMNL-markers (clinical, electrophysiological, imaging and neurofilaments) and their dynamics in ALS has the potential to identify patients early in the course of the disease or even in the asymptomatic stage as better candidates for clinical studies. Furthermore, a risk stratification score should be established to identify the fast progressive ALS patients searching for the factors leading to a more aggressive form of the disease.

In PPMS one can see a trend towards higher neurofilament (NF) level in PMS patients compared to RRMS patients. Similar to ALS, MRI parameters provide also valuable information regarding the process of neurodegeneration in MS. Whole brain atrophy was consistent in all MS patients but with different rates according to clinical phenotypes ranging from 0.5%–0.8% in RRMS to 1.3%–1.4% in SPMS and 1%–1.3% in PPMS [12]. However, other studies showed differences between rates of grey matter atrophy (GMA) and white matter atrophy (WMA) with a relative constant WMA rates (about three-fold the normal) in CIS, RRMS and PMS patients and a much more aggressive GMA in SPMS patients (about 14-fold) when compared to CIS patients (three or four-fold) [108]. Similarly, faster rates of upper cervical cord atrophy was found in SPMS and PPMS patients when compared to RRMS [109].

**Figure 4:**
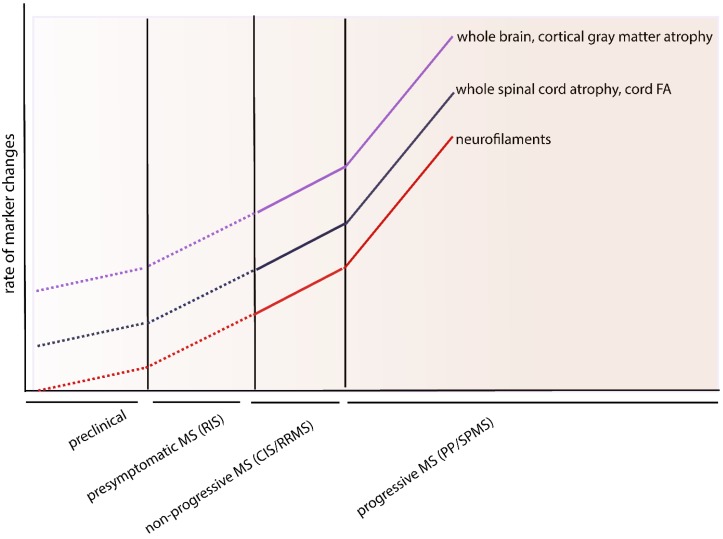
Dynamics of CSF and MRI-parameters of neurodegeneration over the course of the multiple sclerosis (MS). The imaging studies revealed accelerated whole brain, spinal cord and cortical gray matter atrophy in the progressive phase of MS when compared to relapsing remitting multiple sclerosis (RRMS) and clinically isolated syndrome (CIS). Similarly, decrease in cord FA was higher in PPMS compared to RRMS. According to these results, similar changes in the levels of neurofilaments could be postulated.

Furthermore, a statistically significant decrease in cord FA during the course of the disease was seen in PPMS patients [110]. Summarizing these finding one can postulate the following dynamics of neurodegeneration markers in MS-patients (Figure 4).

Here again, like in ALS, the grey matter atrophy is highly progressive with the progression of the disease, compared with the relatively milder white matter atrophy. This highlights the role of gray matter atrophy in different neurodegenerative diseases.

Generally, the studies in ALS showed relatively homogenous results, unlike the PPMS. This could be explained by the differences between PPMS patients regarding the site of lesions, the affection of different functional systems, and the course and duration of disease. While in ALS, the degeneration affects mainly the motor neurons and their projections with disease duration of three to five years followed by death. Thus, postulating and validating a module for the CSF-biomarker dynamics for PPMS would be difficult—Given that until now there is no animal model for the disease to study the presymptomatic or even the preclinical stage. Moreover, little or nothing is known about these stages because of the absence of known risk factors which allow following up the patients in the early stages within the framework of a prospective study. Nonetheless, in a recent follow-up study in patients with radiologically isolated syndrome, 9.6% of the patients (*n* = 14) develop PPMS in five years [111] representing a sample for patients with asymptomatic PPMS for further longitudinal studies regarding dynamics of neurodegenerative markers.

## 7. Conclusions

PPMS and ALS share some common degenerative and inflammatory features involving axonal death, apoptosis and gliosis occurring already in preclinical stages despite their different disease triggering etiologies. Knowledge on mechanisms involved in these pathways will help to develop a strategy to prevent or at least slow the ongoing axonal degeneration and the resulting disability. Validation of CSF biomarkers and analyzing them in conjunction with other markers of neurodegeneration is a promising approach which could be helpful for establishing early diagnostic and prognostic tools, and for assessment of treatment efficacy of disease modifying drugs.

## Abbreviations

ALS: Amyotrophic Lateral Sclerosis; AD: Alzheimer’s disease; BBB: Blood Brain Barrier; CIDP: Chronic Inflammatory Demyelinating Polyneuropathy; CIS: Clinically Isolated Syndrome; CJD: Creutzfeldt Jakob disease; CST: corticospinal tract; EAAT: Excitatory Amino Acid Transporter; ECL: Electrochemiluminescence; EDSS: Expanded Disability Status Scale; ELISA: Enzyme-linked immunosorbent Assay; fALS: Familial ALS; GMA: grey matter atrophy; HSP: Hereditary Spastic Paraplegia; IND: Inflammatory Neurological Diseases; LMNL: Lower Motor Neuron Lesion; MCT: Monocarboxylate Transporter; MEP: motor evoked potentials; MRI: magnetic resonance imaging; MS: Multiple Sclerosis; MSFC: Multiple Sclerosis Functional Composite; MSSS: Multiple Sclerosis Severity Scale; NfH: Neurofilament Heavy-chain; NfL: Neurofilament Light-chain; NIND: Non Inflammatory Neurological Diseases; OND: Other Neurological Diseases; PLS: Primary lateral Sclerosis; PSP: Progressive Supranuclear Palsy; PMS: Progressive Multiple Sclerosis; PPMS: Primary Progressive Multiple Sclerosis; P-tau: Phosphorylated tau; RRMS: Relapsing Remitting Multiple Sclerosis; sALS: Sporadic ALS; SPMS: Secondary Progressive Multiple Sclerosis; t-Tau: total Tau; UMNL: Upper Motor Neuron Lesion.; WMA: white matter atrophy.

## References

[1] Luessi F., Siffrin V., Zipp F. (2012). Neurodegeneration in multiple sclerosis: Novel treatment strategies. Expert Rev. Neurother..

[2] Koch M., Kingwell E., Rieckmann P., Tremlett H., Neurologists U.M.C. (2010). The natural history of secondary progressive multiple sclerosis. J. Neurol. Neurosurg. Psychiatry.

[3] Thompson A.J., Polman C.H., Miller D.H., McDonald W.I., Brochet B., Filippi M.M.X., de Sa J. (1997). Primary progressive multiple sclerosis. Brain.

[4] Frischer J.M., Bramow S., Dal-Bianco A., Lucchinetti C.F., Rauschka H., Schmidbauer M., Laursen H., Sorensen P.S., Lassmann H. (2009). The relation between inflammation and neurodegeneration in multiple sclerosis brains. Brain.

[5] Feinstein A., Freeman J., Lo A.C. (2015). Treatment of progressive multiple sclerosis: What works, what does not, and what is needed. Lancet neurol..

[6] Ferguson T.A., Elman L.B. (2007). Clinical presentation and diagnosis of amyotrophic lateral sclerosis. NeuroRehabilitation.

[7] Vucic S., Rothstein J.D., Kiernan M.C. (2014). Advances in treating amyotrophic lateral sclerosis: Insights from pathophysiological studies. Trends Neurosci..

[8] Traxinger K., Kelly C., Johnson B.A., Lyles R.H., Glass J.D. (2013). Prognosis and epidemiology of amyotrophic lateral sclerosis: Analysis of a clinic population, 1997–2011. Neurol. Clin. Pract..

[9] Neumann M., Sampathu D.M., Kwong L.K., Truax A.C., Micsenyi M.C., Chou T.T., Bruce J., Schuck T., Grossman M., Clark C.M. (2006). Ubiquitinated TDP-43 in frontotemporal lobar degeneration and amyotrophic lateral sclerosis. Science.

[10] Van Den Bosch L., van Damme P., Bogaert E., Robberecht W. (2006). The role of excitotoxicity in the pathogenesis of amyotrophic lateral sclerosis. Biochim. Biophys. Acta.

[11] Rice C.M., Cottrell D., Wilkins A., Scolding N.J. (2013). Primary progressive multiple sclerosis: Progress and challenges. J. Neurol. Neurosurg. Psychiatry.

[12] Martola J., Stawiarz L., Fredrikson S., Hillert J., Bergstrom J., Flodmark O., Kristoffersen Wiberg M. (2007). Progression of non-age-related callosal brain atrophy in multiple sclerosis: A 9-year longitudinal MRI study representing four decades of disease development. J. Neurol. Neurosurg. Psychiatry.

[13] El Mendili M.M., Cohen-Adad J., Pelegrini-Issac M., Rossignol S., Morizot-Koutlidis R., Marchand-Pauvert V., Iglesias C., Sangari S., Katz R., Lehericy S. (2014). Multi-parametric spinal cord MRI as potential progression marker in amyotrophic lateral sclerosis. PLoS ONE.

[14] Faber I., Servelhere K.R., Martinez A.R., D’Abreu A., Lopes-Cendes I., Franca-Jr M.C. (2014). Clinical features and management of hereditary spastic paraplegia. Arq. Neuropsiquiatr..

[15] Gordon P.H., Cheng B., Katz I.B., Pinto M., Hays A.P., Mitsumoto H., Rowland L.P. (2006). The natural history of primary lateral sclerosis. Neurology.

[16] Miller D.H., Leary S.M. (2007). Primary-progressive multiple sclerosis. Lancet Neurol..

[17] Gotkine M., Argov Z. (2007). Clinical differentiation between primary lateral sclerosis and upper motor neuron predominant amyotrophic lateral sclerosis—Author reply. Arch. Neurol..

[18] Gordon P.H. (2013). Amyotrophic lateral sclerosis: An update for 2013 clinical features, pathophysiology, management and therapeutic trials. Aging Dis..

[19] Blokhuis A.M., Groen E.J., Koppers M., van den Berg L.H., Pasterkamp R.J. (2013). Protein aggregation in amyotrophic lateral sclerosis. Acta Neuropathol..

[20] Freischmidt A., Wieland T., Richter B., Ruf W., Schaeffer V., Muller K., Marroquin N., Nordin F., Hubers A., Weydt P. (2015). Haploinsufficiency of TBK1 causes familial ALS and fronto-temporal dementia. Nat. Neurosci..

[21] Lee E.B., Lee V.M., Trojanowski J.Q. (2012). Gains or losses: Molecular mechanisms of TDP43-mediated neurodegeneration. Nat. Rev. Neurosci..

[22] Dormann D., Rodde R., Edbauer D., Bentmann E., Fischer I., Hruscha A., Than M.E., Mackenzie I.R., Capell A., Schmid B. (2010). ALS-associated fused in sarcoma (FUS) mutations disrupt transportin-mediated nuclear import. EMBO J..

[23] Ogawa M., Furukawa Y. (2014). A seeded propagation of Cu, Zn-superoxide dismutase aggregates in amyotrophic lateral sclerosis. Front. Cell. Neurosci..

[24] Lagier-Tourenne C., Polymenidou M., Cleveland D.W. (2010). TDP-43 and FUS/TLS: Emerging roles in RNA processing and neurodegeneration. Hum. Mol. Genet..

[25] Rutherford N.J., Zhang Y.J., Baker M., Gass J.M., Finch N.A., Xu Y.F., Stewart H., Kelley B.J., Kuntz K., Crook R.J. (2008). Novel mutations in TARDBP (TDP-43) in patients with familial amyotrophic lateral sclerosis. PLoS Genet..

[26] Li Y.R., King O.D., Shorter J., Gitler A.D. (2013). Stress granules as crucibles of als pathogenesis. J. Cell Biol..

[27] Zinszner H., Sok J., Immanuel D., Yin Y., Ron D. (1997). TLS (FUS) binds RNA *in vivo* and engages in nucleo-cytoplasmic shuttling. J. Cell Sci..

[28] Dormann D., Haass C. (2011). TDP-43 and FUS: A nuclear affair. Trends Neurosci..

[29] McGeer P.L., McGeer E.G. (2002). Inflammatory processes in amyotrophic lateral sclerosis. Muscle Nerve.

[30] Valori C.F., Brambilla L., Martorana F., Rossi D. (2014). The multifaceted role of glial cells in amyotrophic lateral sclerosis. Cell. Mol. Life Sci..

[31] Zhao W., Beers D.R., Appel S.H. (2013). Immune-mediated mechanisms in the pathoprogression of amyotrophic lateral sclerosis. J. Neuroimmune Pharmacol..

[32] Brites D., Vaz A.R. (2014). Microglia centered pathogenesis in ALS: Insights in cell interconnectivity. Front. Cell. Neurosci..

[33] Lee Y., Morrison B.M., Li Y., Lengacher S., Farah M.H., Hoffman P.N., Liu Y., Tsingalia A., Jin L., Zhang P.W. (2012). Oligodendroglia metabolically support axons and contribute to neurodegeneration. Nature.

[34] Mantovani S., Garbelli S., Pasini A., Alimonti D., Perotti C., Melazzini M., Bendotti C., Mora G. (2009). Immune system alterations in sporadic amyotrophic lateral sclerosis patients suggest an ongoing neuroinflammatory process. J. Neuroimmunol..

[35] Neymotin A., Petri S., Calingasan N.Y., Wille E., Schafer P., Stewart C., Hensley K., Beal M.F., Kiaei M. (2009). Lenalidomide (Revlimid) administration at symptom onset is neuroprotective in a mouse model of amyotrophic lateral sclerosis. Exp. Neurol..

[36] Kang J., Rivest S. (2007). MyD88-deficient bone marrow cells accelerate onset and reduce survival in a mouse model of amyotrophic lateral sclerosis. J. Cell Biol..

[37] Brettschneider J., del Tredici K., Toledo J.B., Robinson J.L., Irwin D.J., Grossman M., Suh E., van Deerlin V.M., Wood E.M., Baek Y. (2013). Stages of pTDP-43 pathology in amyotrophic lateral sclerosis. Ann. Neurol..

[38] Ravits J.M., La Spada A.R. (2009). ALS motor phenotype heterogeneity, focality, and spread: Deconstructing motor neuron degeneration. Neurology.

[39] Ravits J., Appel S., Baloh R.H., Barohn R., Brooks B.R., Elman L., Floeter M.K., Henderson C., Lomen-Hoerth C., Macklis J.D. (2013). Deciphering amyotrophic lateral sclerosis: What phenotype, neuropathology and genetics are telling us about pathogenesis. Amyotroph. Lateral Scler. Front. Degener..

[40] Bruck W. (2005). The pathology of multiple sclerosis is the result of focal inflammatory demyelination with axonal damage. J. Neurol..

[41] Munzel E.J., Williams A. (2013). Promoting remyelination in multiple sclerosis—Recent advances. Drugs.

[42] Patani R., Balaratnam M., Vora A., Reynolds R. (2007). Remyelination can be extensive in multiple sclerosis despite a long disease course. Neuropathol. Appl. Neurobiol..

[43] Patrikios P., Stadelmann C., Kutzelnigg A., Rauschka H., Schmidbauer M., Laursen H., Sorensen P.S., Bruck W., Lucchinetti C., Lassmann H. (2006). Remyelination is extensive in a subset of multiple sclerosis patients. Brain.

[44] Lassmann H., van Horssen J., Mahad D. (2012). Progressive multiple sclerosis: Pathology and pathogenesis. Nat. Rev. Neurol..

[45] Lucchinetti C.F., Bruck W., Rodriguez M., Lassmann H. (1996). Distinct patterns of multiple sclerosis pathology indicates heterogeneity on pathogenesis. Brain Pathol..

[46] Lucchinetti C., Bruck W., Parisi J., Scheithauer B., Rodriguez M., Lassmann H. (2000). Heterogeneity of multiple sclerosis lesions: Implications for the pathogenesis of demyelination. Ann. Neurol..

[47] Hauser S.L., Oksenberg J.R. (2006). The neurobiology of multiple sclerosis: Genes, inflammation, and neurodegeneration. Neuron.

[48] Lassmann H. (2007). Multiple sclerosis: Is there neurodegeneration independent from inflammation?. J. Neurol. Sci..

[49] Fu L., Matthews P.M., de Stefano N., Worsley K.J., Narayanan S., Francis G.S., Antel J.P., Wolfson C., Arnold D.L. (1998). Imaging axonal damage of normal-appearing white matter in multiple sclerosis. Brain.

[50] Tumani H., Hartung H.P., Hemmer B., Teunissen C., Deisenhammer F., Giovannoni G., Zettl U.K., BioMS Study Group (2009). Cerebrospinal fluid biomarkers in multiple sclerosis. Neurobiol. Dis..

[51] Lassmann H. (2014). Mechanisms of white matter damage in multiple sclerosis. Glia.

[52] Davson H., Hollingsworth G., Segal M.B. (1970). The mechanism of drainage of the cerebrospinal fluid. Brain.

[53] Felgenhauer K., Schliep G., Rapic N. (1976). Evaluation of the blood-CSF barrier by protein gradients and the humoral immune response within the central nervous system. J. Neurol. Sci..

[54] Reiber H. (1994). Flow rate of cerebrospinal fluid (CSF)—A concept common to normal blood-CSF barrier function and to dysfunction in neurological diseases. J. Neurol. Sci..

[55] Felgenhauer K. (1995). The filtration concept of the blood-CSF-barrier as basis for the differentiation of CSF proteins. New Concepts of A Blood—Brain Barrier.

[56] Reiber H. (2001). Dynamics of brain-derived proteins in cerebrospinal fluid. Clin. Chim. Acta.

[57] Semra Y.K., Seidi O.A., Sharief M.K. (2002). Heightened intrathecal release of axonal cytoskeletal proteins in multiple sclerosis is associated with progressive disease and clinical disability. J. Neuroimmunol..

[58] Teunissen C.E., Khalil M. (2012). Neurofilaments as biomarkers in multiple sclerosis. Mult. Scler..

[59] Kuhle J., Plattner K., Bestwick J.P., Lindberg R.L., Ramagopalan S.V., Norgren N., Nissim A., Malaspina A., Leppert D., Giovannoni G. (2013). A comparative study of CSF neurofilament light and heavy chain protein in MS. Mult. Scler..

[60] Yabe J.T., Chylinski T., Wang F.S., Pimenta A., Kattar S.D., Linsley M.D., Chan W.K.H., Shea T.B. (2001). Neurofilaments consist of distinct populations that can be distinguished by C-terminal phosphorylation, bundling, and axonal transport rate in growing axonal neurites. J. Neurosci..

[61] Strong M.J. (1999). Neurofilament metabolism in sporadic amyotrophic lateral sclerosis. J. Neurol. Sci..

[62] Fuchs E., Cleveland D.W. (1998). A structural scaffolding of intermediate filaments in health and disease. Science.

[63] Dujmovic I. (2011). Cerebrospinal fluid and blood biomarkers of neuroaxonal damage in multiple sclerosis. Mult. Scler. Int..

[64] Gresle M.M., Shaw G., Jarrott B., Alexandrou E.N., Friedhuber A., Kilpatrick T.J., Butzkueven H. (2008). Validation of a novel biomarker for acute axonal injury in experimental autoimmune encephalomyelitis. J. Neurosci. Res..

[65] Liu Q., Xie F., Siedlak S.L., Nunomura A., Honda K., Moreira P.I., Zhua X., Smith M.A., Perry G. (2004). Neurofilament proteins in neurodegenerative diseases. Cell. Mol. Life Sci..

[66] Munoz D.G., Greene C., Perl D.P., Selkoe D.J. (1988). Accumulation of phosphorylated neurofilaments in anterior horn motoneurons of amyotrophic lateral sclerosis patients. J. Neuropathol. Exp. Neurol..

[67] Figlewicz D.A., Krizus A., Martinoli M.G., Meininger V., Dib M., Rouleau G.A., Julien J.P. (1994). Variants of the heavy neurofilament subunit are associated with the development of amyotrophic lateral sclerosis. Hum. Mol. Genet..

[68] Rosengren L.E., Karlsson J.E., Karlsson J.O., Persson L.I., Wikkelso C. (1996). Patients with amyotrophic lateral sclerosis and other neurodegenerative diseases have increased levels of neurofilament protein in CSF. J. Neurochem..

[69] Brettschneider J., Petzold A., Sussmuth S.D., Ludolph A.C., Tumani H. (2006). Axonal damage markers in cerebrospinal fluid are increased in ALS. Neurology.

[70] Reijn T.S., Abdo W.F., Schelhaas H.J., Verbeek M.M. (2009). CSF neurofilament protein analysis in the differential diagnosis of ALS. J. Neurol..

[71] Zetterberg H., Jacobsson J., Rosengren L., Blennow K., Andersen P.M. (2007). Cerebrospinal fluid neurofilament light levels in amyotrophic lateral sclerosis: Impact of SOD1 genotype. Eur. J. Neurol..

[72] Tortelli R., Ruggieri M., Cortese R., D'Errico E., Capozzo R., Leo A., Mastrapasqua M., Zoccolella S., Leante R., Livrea P. (2012). Elevated cerebrospinal fluid neurofilament light levels in patients with amyotrophic lateral sclerosis: A possible marker of disease severity and progression. Eur.J. Neurol.

[73] Boylan K.B., Glass J.D., Crook J.E., Yang C., Thomas C.S., Desaro P., Johnston A., Overstreet K., Kelly C., Polak M. (2013). Phosphorylated neurofilament heavy subunit (pNF-H) in peripheral blood and csf as a potential prognostic biomarker in amyotrophic lateral sclerosis. J. Neurol. Neurosurg. Psychiatry.

[74] Lu C.H., Macdonald-Wallis C., Gray E., Pearce N., Petzold A., Norgren N., Giovannoni G., Fratta P., Sidle K., Fish M. (2015). Neurofilament light chain: A prognostic biomarker in amyotrophic lateral sclerosis. Neurology.

[75] Kuhle J., Regeniter A., Leppert D., Mehling M., Kappos L., Lindberg R.L., Petzold A. (2010). A highly sensitive electrochemiluminescence immunoassay for the neurofilament heavy chain protein. J. Neuroimmunol..

[76] Petzold A., Eikelenboom M.J., Keir G., Grant D., Lazeron R.H., Polman C.H., Uitdehaag B.M., Thompson E.J., Giovannoni G. (2005). Axonal damage accumulates in the progressive phase of multiple sclerosis: Three year follow up study. J. Neurol. Neurosurg. Psychiatry.

[77] Romme Christensen J., Bornsen L., Khademi M., Olsson T., Jensen P.E., Sorensen P.S., Sellebjerg F. (2013). CSF inflammation and axonal damage are increased and correlate in progressive multiple sclerosis. Mult. Scler..

[78] Eikelenboom M.J., Petzold A., Lazeron R.H., Silber E., Sharief M., Thompson E.J., Barkhof F., Giovannoni G., Polman C.H., Uitdehaag B.M. (2003). Multiple sclerosis: Neurofilament light chain antibodies are correlated to cerebral atrophy. Neurology.

[79] Salzer J., Svenningsson A., Sundstrom P. (2010). Neurofilament light as a prognostic marker in multiple sclerosis. Mult. Scler..

[80] Madeddu R., Farace C., Tolu P., Solinas G., Asara Y., Sotgiu M.A., Delogu L.G., Prados J.C., Sotgiu S., Montella A. (2013). Cytoskeletal proteins in the cerebrospinal fluid as biomarker of multiple sclerosis. Neurol. Sci..

[81] Shahani N., Brandt R. (2002). Functions and malfunctions of the tau proteins. Cell. Mol. Life Sci..

[82] Jimenez-Jimenez F.J., Hernanz A., Medina-Acebron S., de Bustos F., Zurdo J.M., Alonso H., Puertas I., Barcenilla B., Sayed Y., Cabrera-Valdivia F. (2005). Tau protein concentrations in cerebrospinal fluid of patients with amyotrophic lateral sclerosis. Acta Neurol. Scand..

[83] Paladino P., Valentino F., Piccoli T., Piccoli F., La Bella V. (2009). Cerebrospinal fluid tau protein is not a biological marker in amyotrophic lateral sclerosis. Eur.J. Neurol..

[84] Grossman M., Elman L., McCluskey L., McMillan C.T., Boller A., Powers J., Rascovsky K., Hu W., Shaw L., Irwin D.J. (2014). Phosphorylated tau as a candidate biomarker for amyotrophic lateral sclerosis. JAMA Neurol..

[85] Sussmuth S.D., Tumani H., Ecker D., Ludolph A.C. (2003). Amyotrophic lateral sclerosis: Disease stage related changes of tau protein and S100 β in cerebrospinal fluid and creatine kinase in serum. Neurosci. Lett..

[86] Kapaki E., Paraskevas G.P., Michalopoulou M., Kilidireas K. (2000). Increased cerebrospinal fluid tau protein in multiple sclerosis. Eur. Neurol..

[87] Brettschneider J., Maier M., Arda S., Claus A., Sussmuth S.D., Kassubek J., Tumani H. (2005). Tau protein level in cerebrospinal fluid is increased in patients with early multiple sclerosis. Mult. Scler..

[88] Terzi M., Birinci A., Cetinkaya E., Onar M.K. (2007). Cerebrospinal fluid total tau protein levels in patients with multiple sclerosis. Acta Neurol. Scand..

[89] Bartosik-Psujek H., Archelos J.J. (2004). Tau protein and 14-3-3 are elevated in the cerebrospinal fluid of patients with multiple sclerosis and correlate with intrathecal synthesis of IgG. J. Neurol..

[90] Guimaraes I., Cardoso M.I., Sa M.J. (2006). Tau protein seems not to be a useful routine clinical marker of axonal damage in multiple sclerosis. Mult. Scler..

[91] Colucci M., Roccatagliata L., Capello E., Narciso E., Latronico N., Tabaton M., Mancardi G.L. (2004). The 14-3-3 protein in multiple sclerosis: A marker of disease severity. Mult. Scler..

[92] Anderson J.M., Patani R., Reynolds R., Nicholas R., Compston A., Spillantini M.G., Chandran S. (2010). Abnormal tau phosphorylation in primary progressive multiple sclerosis. Acta Neuropathol..

[93] Downing K.H. (2000). Structural basis for the interaction of tubulin with proteins and drugs that affect microtubule dynamics. Annu. Rev. Cell Dev. Biol..

[94] Laferriere N.B., MacRae T.H., Brown D.L. (1997). Tubulin synthesis and assembly in differentiating neurons. Biochem. Cell Biol..

[95] Gunning P., Hardeman E., Jeffrey P., Weinberger R. (1998). Creating intracellular structural domains: Spatial segregation of actin and tropomyosin isoforms in neurons. Bioessays.

[96] Doussau F., Augustine G.J. (2000). The actin cytoskeleton and neurotransmitter release: An overview. Biochimie.

[97] Tumani H., Teunissen C., Süssmuth S., Otto M., Ludolph A.C., Brettschneider J. (2008). Cerebrospinal fluid biomarkers of neurodegeneration in chronic neurological diseases. Expert Rev. Mol. Diagn..

[98] Vinsant S., Mansfield C., Jimenez-Moreno R., del Gaizo Moore V., Yoshikawa M., Hampton T.G., Prevette D., Caress J., Oppenheim R.W., Milligan C. (2013). Characterization of early pathogenesis in the SOD1(G93A) mouse model of ALS: Part II, results and discussion. Brain Behav..

[99] Vinsant S., Mansfield C., Jimenez-Moreno R., del Gaizo Moore V., Yoshikawa M., Hampton T.G., Prevette D., Caress J., Oppenheim R.W., Milligan C. (2013). Characterization of early pathogenesis in the SOD1(G93A) mouse model of ALS: Part I, background and methods. Brain Behav..

[100] De Carvalho M., Swash M. (2006). The onset of amyotrophic lateral sclerosis. J. Neurol. Neurosurg. Psychiatry.

[101] Aggarwal A., Nicholson G. (2001). Normal complement of motor units in asymptomatic familial (SOD1 mutation) amyotrophic lateral sclerosis carriers. J. Neurol. Neurosurg. Psychiatry.

[102] Magnus T., Beck M., Giess R., Puls I., Naumann M., Toyka K.V. (2002). Disease progression in amyotrophic lateral sclerosis: Predictors of survival. Muscle Nerve.

[103] Agosta F., Valsasina P., Riva N., Copetti M., Messina M.J., Prelle A., Comi G., Filippi M. (2012). The cortical signature of amyotrophic lateral sclerosis. PLoS ONE.

[104] Verstraete E., van den Heuvel M.P., Veldink J.H., Blanken N., Mandl R.C., Hulshoff Pol H.E., van den Berg L.H. (2010). Motor network degeneration in amyotrophic lateral sclerosis: A structural and functional connectivity study. PLoS ONE.

[105] Mezzapesa D.M., D’Errico E., Tortelli R., Distaso E., Cortese R., Tursi M., Federico F., Zoccolella S., Logroscino G., Dicuonzo F. (2013). Cortical thinning and clinical heterogeneity in amyotrophic lateral sclerosis. PLoS ONE.

[106] Menke R.A., Korner S., Filippini N., Douaud G., Knight S., Talbot K., Turner M.R. (2014). Widespread grey matter pathology dominates the longitudinal cerebral MRI and clinical landscape of amyotrophic lateral sclerosis. Brain.

[107] Kwan J.Y., Meoded A., Danielian L.E., Wu T., Floeter M.K. (2012). Structural imaging differences and longitudinal changes in primary lateral sclerosis and amyotrophic lateral sclerosis. Neuroimage Clin..

[108] Fisher E., Lee J.C., Nakamura K., Rudick R.A. (2008). Gray matter atrophy in multiple sclerosis: A longitudinal study. Ann. Neurol..

[109] Lukas C., Knol D.L., Sombekke M.H., Bellenberg B., Hahn H.K., Popescu V., Weier K., Radue E.W., Gass A., Kappos L. (2014). Cervical spinal cord volume loss is related to clinical disability progression in multiple sclerosis. J. Neurol. Neurosurg. Psychiatry.

[110] Agosta F., Absinta M., Sormani M.P., Ghezzi A., Bertolotto A., Montanari E., Comi G., Filippi M. (2007). *In vivo* assessment of cervical cord damage in MS patients: A longitudinal diffusion tensor MRI study. Brain.

[111] Okuda D.T., Siva A., Kantarci O., Inglese M., Katz I., Tutuncu M., Keegan B.M., Donlon S., le Hua H., Vidal-Jordana A. (2014). Radiologically isolated syndrome: 5-Year risk for an initial clinical event. PLoS ONE.

